# Editorial: Mechanisms regulating immune evasion by B-cell lymphoma

**DOI:** 10.3389/fimmu.2026.1912492

**Published:** 2026-06-29

**Authors:** Nethaji Muniraj, Azizul Haque

**Affiliations:** 1 Children’s National Hospital, Center for Cancer and Immunology Research (CCIR), Washington, DC, United States; 2 Department of Pharmacology and Immunology, and Neurosurgery, Medical University of South Carolina (MUSC), Charleston, SC, United States; 3 Hollings Cancer Center, Medical University of South Carolina (MUSC), Charleston, SC, United States; 4 Ralph H. Johnson Veterans Affairs (VA) Medical Center, Charleston, SC, United States

**Keywords:** B-cell lymphoma, diffuse large B-cell lymphoma (DLBCL), immune checkpoint, immune evasion, immunotherapy, tumor microenvironment

## Introduction

B-cell lymphomas are a heterogeneous group of hematologic malignancies that arise from B lymphocytes at different stages of differentiation. They encompass a broad spectrum of diseases, ranging from indolent forms such as follicular lymphoma and chronic lymphocytic leukemia to highly aggressive entities including diffuse large B-cell lymphoma (DLBCL) and Burkitt lymphoma. Despite advances in immunotherapy, targeted therapy, and cell-based approaches, many patients experience relapses or develop treatment resistance, underscoring the need for a deeper understanding of the biological mechanisms driving lymphoma progression.

**Figure 1:**
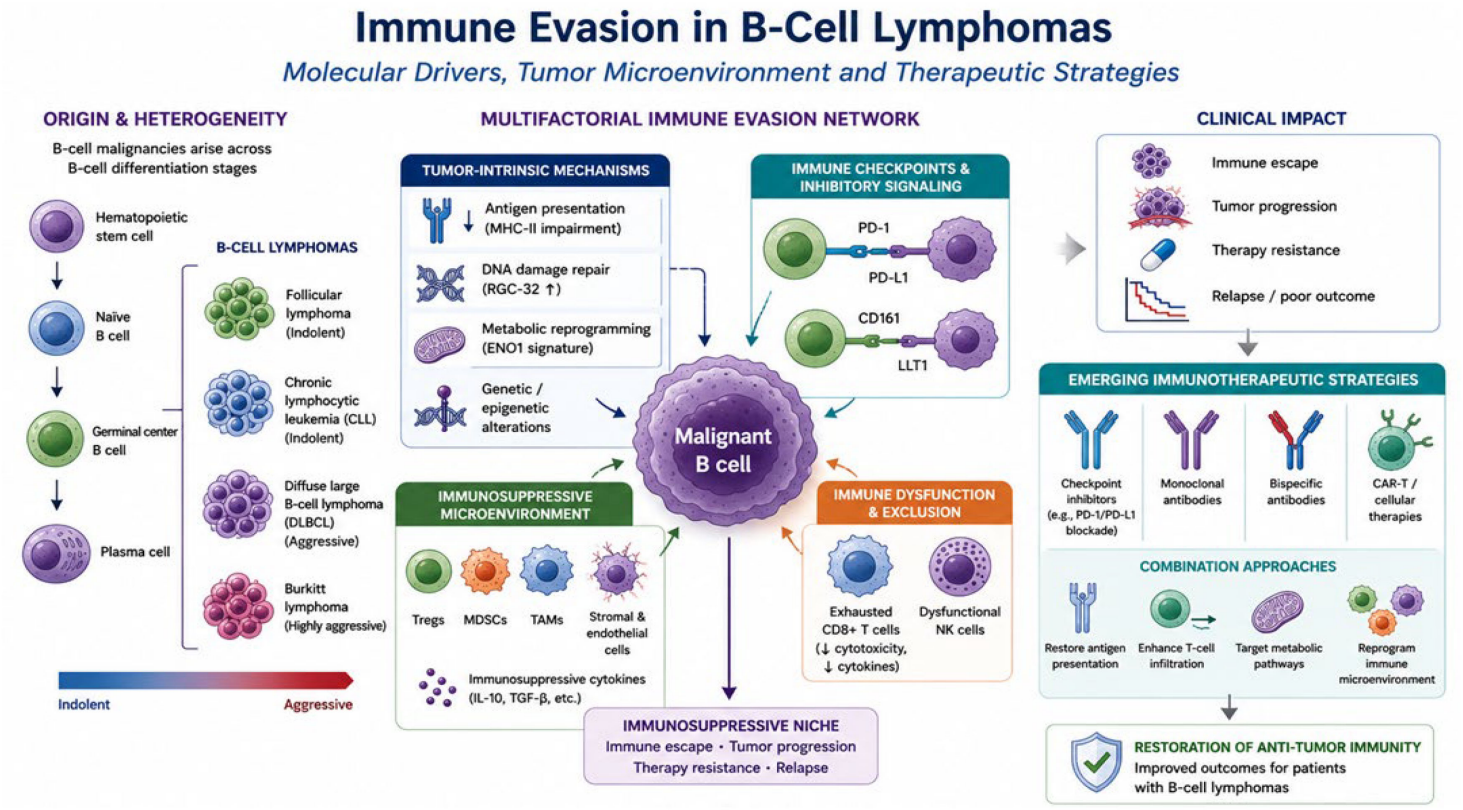
B-cell lymphomas represent a heterogeneous group of malignancies that evade immune surveillance through a complex interplay of tumor-intrinsic and microenvironmental mechanisms. Malignant B cells employ strategies such as impaired antigen presentation, immune checkpoint activation, metabolic reprogramming, and recruitment of immunosuppressive cells to establish an immune-evasive niche. These processes promote tumor progression, immune dysfunction, and therapeutic resistance. Advances in genomic and immune profiling have identified key pathways and biomarkers that inform emerging therapeutic strategies, including checkpoint blockades, cellular therapies, and combination approaches aimed at restoring effective anti-tumor immunity.

A hallmark of B-cell lymphomas is their ability to evade immune surveillance. Under normal physiological conditions, the immune system continuously recognizes and eliminates transformed cells through the coordinated action of innate and adaptive immune responses. However, malignant B cells acquire multiple strategies to escape immune recognition and destruction by the immune system. These strategies include impaired antigen presentation, dysregulation of immune checkpoint pathways, secretion of immunosuppressive cytokines, recruitment of regulatory immune cell populations, metabolic reprogramming, and remodeling of the tumor microenvironment. Together, these alterations create an immunosuppressive niche that supports tumor growth, promotes therapeutic resistance, and contributes to disease progression (**Figure 1**).

Recent advances in genomic sequencing, immune profiling, and single-cell technologies have significantly expanded our understanding of the complex interactions between malignant B cells and the surrounding immune microenvironment. These findings have elucidated novel mechanisms of immune evasion and identified promising therapeutic targets for enhancing anti-tumor immunity.

This thematic study series, titled “Mechanisms of Immune Evasion in B-Cell Lymphomas,” was compiled to shed light on new data regarding the molecular and cellular factors—as well as microenvironmental characteristics—that enable various B-cell malignancies to evade the immune response. The Research Topic comprises original research articles, reviews, and case reports that collectively deepen the understanding of lymphoma biology, mechanisms of therapy resistance, prognostic biomarkers, and immunotherapeutic strategies.

## Molecular drivers of immune evasion and therapeutic resistance

The ability of malignant B cells to evade immune surveillance is determined not only by extrinsic influences from the tumor microenvironment but also by intrinsic molecular alterations that promote tumor survival, immune cell exclusion, and therapeutic resistance. Increasing evidence suggests that genetic, epigenetic, and metabolic changes within lymphoma cells directly influence anti-tumor immune responses and contribute to disease progression.

Several studies addressing this Research Topic provide significant insights into these tumor-specific mechanisms. Zhang et al. investigated the role of response gene to complement 32 (RGC-32) in DLBCL and demonstrated its involvement in promoting tumorigenesis through enhanced DNA damage repair. Importantly, their findings show that the elevated RGC-32 expression was associated with reduced CD8+ T-cell infiltration, suggests that RGC-32 contributes not only to tumor survival but also to the immune exclusion.

Metabolic reprogramming has emerged as another key factor determining lymphoma progression and immune regulatory processes. Yan et al. developed an ENO1-related gene signature that enables the prediction of disease course and treatment response in DLBCL. Their findings reinforce the growing understanding that metabolic pathways influence both tumor biology and immune cell function. Consequently, the identification of metabolism-associated biomarkers may therefore provide new opportunities for patient stratification and therapeutic intervention.

Treatment resistance remains a major challenge in the management of aggressive B-cell lymphomas. Yang et al. conducted an in-depth analysis of the mechanisms underlying resistance to first-line treatment in DLBCL. Their study highlights how a combination of factors—including genetic heterogeneity, signaling pathway dysregulation, immune escape mechanisms, and interactions with the microenvironment—contributes to treatment failure. These findings underscore the need for combination therapeutic strategies capable of targeting both lymphoma-intrinsic survival pathways and the immunosuppressive tumor microenvironment.

Overall, these studies demonstrate that immune escape in B-cell lymphomas is a multifactorial process involving a complex interplay between molecular signaling pathways, DNA repair mechanisms, metabolic adaptations, and resistance-associated networks.

## Tumor microenvironment and immune regulation

The tumor microenvironment plays a key role in the pathogenesis and progression of lymphoma. Beyond alterations within the malignant cells themselves, interactions between lymphoma cells and surrounding immune, stromal, and endothelial components significantly influence disease characteristics and treatment responses.

Several articles in this Research Topic highlight the complexity of these interactions. The review article by Yichu Fu and colleagues showed the important insights into immune activation and microenvironmental crosstalk that support tumor persistence and immune dysfunction. Their findings underscore the significance of alterations in immunological signaling pathways for disease progression and identify potential therapeutic targets.

The interaction between immunomodulatory receptors and their ligands represents another critical mechanism of immune evasion. Research conducted by Rjoop et al., which investigated the expression of CD161 and “lectin-like transcript 1” (LLT1) in Hodgkin lymphoma, expands our understanding of immunomodulatory signaling pathways within the lymphoma microenvironment. The identification of these molecules on Reed-Sternberg cells suggests the existence of a previously underappreciated pathway by which malignant cells suppress anti-tumor immune responses and evade immune surveillance.

Increasing attention is being paid to issues of immunoregulation, as evidence indicates that lymphoma cells create an immunosuppressive microenvironment by recruiting regulatory T cells, myeloid-derived suppressor cells, and tumor-associated macrophages. At the same time, effector T cells and natural killer (NK) cells often exhibit signs of functional exhaustion, characterized by reduced cytotoxicity and diminished cytokine production. Collectively, these changes suppress the antitumor immune response and contribute to disease progression.

Furthermore, advancements in genomic and immunological profiling technologies are new insights into the possibilities for understanding the heterogeneity of lymphoma. Studies investigating prognostic risk groups for IgM gammopathy has identified genomic and immunological biomarkers that enable a more comprehensive assessment of potential immune factors influencing the disease course and clinical outcomes, beyond the MYD88-L265P mutation.

Collectively, these studies underscore the pivotal role of the tumor microenvironment in modulating immune evasion mechanisms across various B-cell malignancies and highlight the importance of strategies aimed at restoring effective anti-tumor immunity.

## Clinical implications and emerging immunotherapeutic strategies

A growing understanding of the mechanisms by which B-cell lymphomas evade the immune response has significantly influenced the development of new therapeutic approaches. Immunotherapy which includes monoclonal antibodies, immune checkpoint inhibitors, bispecific antibodies, and cellular therapies transformed the treatment of many hematologic malignancies. Nevertheless, the response to therapy remains variable, reflecting the complexity and heterogeneity of the mechanisms underlying tumor evasion of immune surveillance.

The clinical relevance of immune checkpoint pathways is highlighted by the case report describing a durable response to PD-1 blockade in ALK-positive large B-cell lymphoma following failure of targeted therapy and conventional chemoimmunotherapy. This observation demonstrates the potential of immune checkpoint inhibition in certain lymphoma subtypes and suggests the importance of molecular and immunological profiling when devising therapeutic strategies.

Similarly, the report of Epstein-Barr virus-positive follicular lymphoma with high PD-L1 expression illustrates the interplay between viral oncogenesis and immune regulation. The observation of elevated PD-L1 expression supports the role of checkpoint pathways in mediating immune escape and suggests potential utility for immune checkpoint-targeted therapies in selected patients.

Beyond checkpoint inhibition, the studies included in this Research Topic collectively suggest that effective lymphoma therapy may require multifaceted approaches targeting both tumor-intrinsic and microenvironmental mechanisms of immune evasion. Therapeutic strategies aimed at restoring antigen presentation, enhancing T-cell infiltration, reversing immune exhaustion, modulating metabolic pathways, and disrupting immunosuppressive cellular networks may provide synergistic benefits when combined with existing treatment strategies.

## Future perspectives and conclusions

The studies assembled in this Research Topic collectively demonstrate that immune evasion is a fundamental hallmark of B-cell lymphomas and a major determinant of disease progression, therapeutic resistance, and clinical outcome. While significant advances have been made in characterizing the molecular and cellular mechanisms that enable malignant B cells to escape immune surveillance, many questions remain regarding the dynamic interactions between lymphoma cells and the immune microenvironment.

Several recurring themes emerge from this Research Topic. First, immune evasion is driven by both tumor-intrinsic and tumor-extrinsic mechanisms. Second, the tumor microenvironment plays an important role in shaping immune responses through immune checkpoints, cytokine networks, regulatory immune cells, and stromal interactions. Third, advances in genomic and immune profiling technologies are increasingly enabling the identification of biomarkers that can improve patient stratification and guide personalized therapeutic approaches.

The growing success of immunotherapeutic strategies highlights the translational importance of understanding immune escape mechanisms. Nevertheless, treatment resistance remains a significant challenge, emphasizing the need for rational combination therapies that simultaneously target multiple pathways involved in immune suppression. Future research should focus on integrating molecular, immunological, and clinical data to better define disease heterogeneity and identify novel therapeutic vulnerabilities.

In conclusion, the articles presented in this Research Topic provide valuable insights into the diverse mechanisms regulating immune evasion in B-cell lymphomas. Together, they advance our understanding of lymphoma immunobiology, identify potential biomarkers and therapeutic targets, and highlight emerging opportunities for improving patient care. We hope that this Research Topic will stimulate further investigation and foster the development of innovative strategies capable of restoring effective anti-tumor immunity and ultimately improving outcomes for patients with B-cell malignancies.

